# Is there evidence that emotional reasoning processing underlies emotional disorders in adults? A systematic review

**DOI:** 10.1007/s12144-022-03884-4

**Published:** 2022-11-09

**Authors:** Macarena Paredes-Mealla, Verónica Martínez-Borba, Marta Miragall, Azucena García-Palacios, Rosa Mª Baños, Carlos Suso-Ribera

**Affiliations:** 1 Department of Basic and Clinical Psychology and Psychobiology, Jaume I University, Avda. Vicent Sos Baynat s/n, 12071 Castellon de la Plana, Spain; 2 Department of Personality, Evaluation and Psychological Treatment, University of Valencia, Valencia, Spain; 3 CIBER Fisiopatología Obesidad y Nutrición, Instituto Carlos III, Madrid, Spain; 4 Instituto Polibienestar, University of Valencia, Valencia, Spain

**Keywords:** Emotional reasoning, Transdiagnostic, Emotional disorders, Anxiety, Depression

## Abstract

The prevalence of emotional disorders has increased in recent times. Emotional Reasoning (ER), which is a transdiagnostic process, occurs when feelings, rather than objective evidence, are used as a source of information to make judgements about the valence of a situation. Differences in ER may explain the existence and maintenance of emotional disorders. The objective is to systematically review the role of ER in the occurrence and severity of emotional disorders. Following PRISMA guidelines, we searched through: PubMed, PsycInfo, Scopus and The Cochrane Library. Search terms were "Emotional Reasoning", "ex-consequentia reasoning", "Affect-as-information"; and "emotional disorders", "anxiety", "depression", "depressive". Nine articles were included. An association was demonstrated between ER and a greater degree of anxious symptomatological severity. In depressive symptomatology, no significant differences were found. One study reported the effect of Cognitive Behavioural Therapy on ER bias, finding no changes after the intervention. Finally, another study evaluated the efficacy of computerised experiential training in reducing ER bias, showing significant differences. There are few studies on ER and its evolution in research has not been uniform over time. Encouragingly, though, research to date suggests that ER is a transdiagnostic process involved in several anxiety disorders. More investigation is needed to dilucidate whether ER also underlies the onset and maintenance of depressive disorders.

## Introduction

Emotional disorders, which include depressive and anxiety disorders, along with related disorders such as borderline personality disorder, insomnia disorder, eating disorders, persistent complex bereavement disorder, obsessive–compulsive disorder, and posttraumatic stress disorder (Bullis et al., 2019), have become one of the leading causes of disability worldwide (Friedrich, 2017; Stein & Craske, 2017; Steinhauser et al., 2017). Specifically, depressive and related disorders have been suggested to affect between 13.1% and 15.6% of individuals globally (James et al., 2018), while the lifetime prevalence of anxiety disorders, such as separation anxiety disorder, selective mutism, specific phobia, social anxiety disorder (SAD), panic disorder, agoraphobia, generalized anxiety disorder (GAD), or substance induced anxiety disorder, has been estimated to range from 4.8% to 10.9% (Stein et al., 2017). Also importantly, these numbers have alarmingly increased during the COVID-19 pandemic, both in the case of depression (33.7%) and anxiety disorders (31.9%; Salari et al., 2020). Thus, adequate management of these mental health problems is an urge.

Traditionally, each emotional disorder (e.g., social phobia, agoraphobia, or generalized anxiety) has been targeted with a psychological intervention specifically addressed for that problem. The specific-disorder approach, however, has some challenges. For example, having a specific intervention for each emotional disorder is expensive as it requires training therapists in numerous specific treatments. In addition, it makes group therapy, which is a more cost-effective alternative to individual therapy (Tucker & Oei, 2006), challenging due to the need to recruit patients with the same disorder (Barlow et al., 2017; Chisholm et al., 2016; Rodriguez-Seijas et al., 2017). The global net present value of investment needed to treat depression and anxiety over the period 2016–30 has been estimated to be US$147 billion (Chisholm et al., 2016). Therefore, cost-effective interventions (e.g., online and group-based) are an urge. Another challenge of specific interventions for each emotional disorder lies in the high comorbidity rates between emotional disorders. It has been argued that presenting one comorbid emotional disorder occurs in more than 75% of cases (Barlow et al., 2017; González-Robles et al., 2018; Steele et al., 2018). As a result of this, several authors have suggested that shared processes exist between emotional disorders and have argued that comorbidities would be explained by these common underlying processes between disorders (Barlow et al., 2017). Because of these shared processes, emotional disorders could be more effectively addressed with a single transdiagnostic intervention as opposed to specific treatments for each emotional disorder.

The transdiagnostic perspective, an alternative to this 'disorder-focused' approach to care in emotional problems, emerged some years ago (Barlow et al., 2004). Different to the disease-specific approach to emotional disorders, the transdiagnostic perspective focuses on what the disorders have in common (Harvey et al., 2004). Most specifically, the transdiagnostic approach places the emphasis on the processes involved in predisposing, precipitating, and perpetuating the development of a disorder (Bullis et al., 2019). Transdiagnostic interventions have been now effectively implemented in persons with both anxious and depressive symptomatology in different forms, including individual (Sakiris & Berle, 2019), group (Laposa et al., 2017; Talkovsky et al., 2017), or even online format (Díaz-García et al., 2021; Kladnitski et al., 2020; Weisel et al., 2019). They have also been implemented both in private (Bullis et al., 2014) and public settings (Osma et al., 2021) with excellent results.

While acknowledging the treatment benefits obtained by transdiagnostic approaches, there are some important challenges that still need to be addressed by researchers interested in the transdiagnostic management of emotional disorders. An example of this is the determination of factors or processes that are common across emotional disorder (transdiagnostic), as opposed to those that define the specific clinical characteristics of different clinical entities. The identification of transdiagnostic processes has important implications (Harvey et al., 2004, 2011). First, it allows to explain the diagnostic overlap between different categories or mental disorders (disorders that are likely to be comorbid because they share common processes). Second, it facilitates transferring the advances made for a particular disorder to other disorders. Finally, it makes it easier to design treatment strategies that are effective for a wide range of disorders.

Regarding these transdiagnostic processes, Harvey et al. (2004) model is one of the most ambitious and comprehensive proposals to date. After reviewing empirical studies, the authors grouped the 14 transdiagnostic processes evidenced in the literature into 5 domains or “key processes”: attention (external selective attention, internal selective attention, and attentional avoidance); memory (explicit selective memory, recurrent memory, and overgeneralized memory); reasoning (interpretative biases, expectation biases, and emotional reasoning); thinking (recurrent negative thinking, positive and negative metacognitive beliefs, and thought suppression); and behavior (avoidance and safety behaviors).

In this review, we will focus on the Reasoning domain and, more specifically, on the transdiagnostic process named Emotional Reasoning (ER) because it is one of the least investigated, yet promising, processes in the transdiagnostic approach. An ER heuristic occurs when feelings, rather than objective evidence, are used as a source of information when making evaluative judgements about the external world (‘How do I feel about it?Arntz et al., 1995; Schwarz & Clore, 1983). For example, a person with high ER might think "if I feel anxious, that means there must be danger" instead of "If there is danger, it is normal that I feel anxious" (Arntz et al., 1995). Alloy and Abramson (1988) argued that reasoning bias occurs when thinking about the world is based on the emotion that the person feels, which tends to lead to lead to certain conclusions in a systematic and regular manner, both across time and across different contexts. The authors argued that reasoning biases could occur at several levels, that is, when making interpretations, when inferring the causes of events (attributional reasoning), and when judging the likelihood or expectancies of events. The authors suggested that these were all likely to be important for emotional disorders.

An extensive ER literature has found that mood can provide valuable information when performing a task or interacting with the environment (Clore, 1992; Clore & Bar-Anan, 2007; Mancini et al., 2008; Schwarz & Clore, 2003; Scott & Cervone, 2002; Watkins & Mason, 2002). Heuristics are indeed valuable in situations in which an individual’s emotions or intuition can assist in making quick and effective decisions. However, ER, like other heuristics, can also provide misguided results (Harvey et al., 2004). In this case, when the ER heuristic repeatedly gives rise to erroneous interpretations, it might contribute to excessive emotional negative states and psychopathology (e.g., anxiety sensitivity might lead individuals to infer danger when they experience anxiety, so emotional reasoning might cause people to fear anxiety symptoms) (Berle & Moulds, 2013b). Several studies support the existence of biases in ER in persons with different emotional disorders (Arntz et al., 1995; Lommen et al., 2013; Verwoerd et al., 2016). However, the literature is this topic is still poorly structured and the extent to which ER has been reliably associated with the occurrence and severity of different emotional disorders is unclear.

The main goal of this study is to systematically review the evidence regarding the role of ER in emotional disorders. In particular, the focus will be on exploring the extent to which the literature suggests that ER is involved in the occurrence and severity of different emotional disorders (anxiety-related disorders and depression). With this goal in mind, we used the Preferred Reporting Items for Systematic Reviews and Meta-Analyses (PRISMA; Page et al., 2021) to identify, select, and critically appraise relevant research while minimizing bias. The first section of the review summarizes the results reported for each clinical group (anxiety-related disorders and depression). In the second section we provide a critical discussion of the results, focusing on the differences between clinical groups, along with the scope and limitations of the assessment tools used to date. Finally, we present recommendations for future research to help advance the field.

## Methods

### Search strategy

The search was conducted through four databases: PubMed, PsycInfo, Scopus, and The Cochrane Library. Attending to previous recommendations when conducting systematic reviews, we selected specific databases according to the field of study (Perestelo-Perez, 2013). The present review was registered at PROSPERO and the protocol can be found at: (https://www.crd.york.ac.uk/prospero/display_record.php?ID=CRD42020183308) Key search terms were divided in two groups: those related with the transdiagnostic process (“Emotional Reasoning”, “ex-consequentia reasoning”, “Affect-as-information”) and terms associated with mental health (“emotional disorders”, “anxiety”, “depression”, “depressive”. See Appendix A for the complete list of search terms and combinations. Results containing these words in the title, abstract, or keywords were considered for further inspection. No date, language, or publication status restrictions were set. The first search was conducted on May 12, 2020 and then again in September 1, 2022, to include recently published studies. The PRISMA guidelines (PRISMA Page et al., 2021); were followed to conduct the present review.

### Study selection

Eligibility criteria included studies evaluating adults with emotional disorders in which ER was specifically assessed. The following diagnoses were included according to a recent definition of emotional disorders (Bullis et al., 2019): Depressive disorders, anxiety disorders (such as separation anxiety disorder, selective mutism, specific phobia, social anxiety disorder (SAD), panic disorder, agoraphobia, generalized anxiety disorder, or substance induced anxiety disorder), borderline personality disorder, insomnia disorder, eating disorders, persistent complex grief disorder, obsessive–compulsive disorder, or post-traumatic stress disorder. Studies with children were excluded, as well as research with patients with neurodevelopmental disorders, schizophrenia spectrum, psychotic disorders, bipolar disorder and related disorders, dissociative disorders, and somatic symptom disorders.

**Fig. 1:**
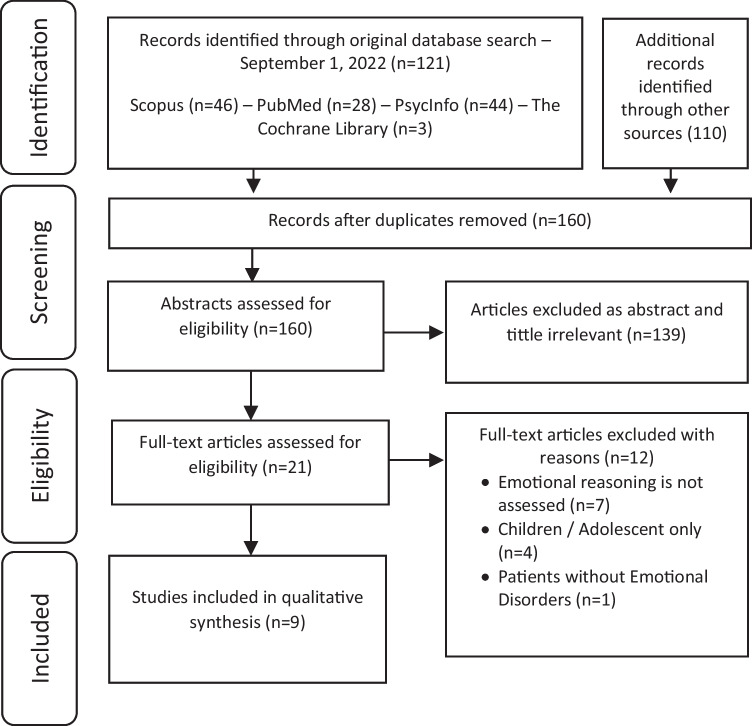
Flow diagram of study selection following PRISMA guidelines

During the first search across the four data bases, 121 results were found. To make sure that no article was left out, an additional search was made based on the bibliographic references of relevant articles and the corresponding authors were contacted. This resulted in 110 articles. After eliminating 71 duplicates, the remaining 160 records were screened attending to the title and abstract. We removed 139 records because they were not considered to be relevant after an initial screening. Of the remaining 21 articles, 12 were removed because they did not meet eligibility criteria after reading the full text. See Fig. 1 for the flow diagram of study selection. The search and screening of articles were performed independently by the first two authors (MPM and VMB). When authors disagreed, they met to discuss the case taking into account each of the eligibility criteria to determine eligibility. The initial inter-rater agreement was calculated (Cohen’s kappa). This coefficient showed a substantial agreement, represented by a kappa of 0.872 (SD = 0.089; 95% CI, 0.697, 1.000). If agreement was not met, a third author was consulted (CSR). Finally, 9 articles were included in the review.

### Data extraction

Data were collected on the socio-demographic characteristics of the participants (age and sex), the sample size according to the condition, the type of psychopathology, the assessment measures used (ER and other variables), the type of treatment implemented (if existent), and the key study findings. Two people extracted the data independently. When the reviewers disagreed, they met to discuss the case. Detailed information from the final group of articles was maintained in an Excel spreadsheet (see Table 1 for the characteristics of the included studies).

**Table 1:** Characteristics of the included studies

Reference-Country	Sample -Methodological design	Psychological Disorder	Sources of construct validity	ER Task / Training characteristics	Key Findings
Arntz et al., (1995)- The Netherlands	133 (Clinical *n* = 119; Non-clinical *n* = 24). Age and sex not reported. Case-Control study	Spider phobia (*n* = 9) Panic disorder (*n* = 41) Social phobia (*n* = 38) Other: OCD (*n* = 13) GAD (*n* = 5), PTSD (*n* = 5), Simple phobia (not spider phobia; *n* = 5), A-typical anxiety disorder (*n* = 3)	Structured Clinical Interview for the DSM-III-R SCL-90	- Administration: Paper and pencil. - Number of scenarios: 4 (spider phobia, panic disorder relevant, social phobia and control situation). - Assessment: 100mm VAS (1 dependent variable: dangerous; and 5 additional outcomes: safety, control, unpleasantness and good vs bad expected outcome).	- All ED groups showed a danger inflating effect of the anxiety response information (*t* > 3.09 and *p* < .002 for all within group effects). Anxiety patients were not only influenced by objective danger information, but also by anxiety response information [*F* (4,174) = 3.73, *p* < 0.001]. - Healthy controls were not influenced by anxiety response information [*t* (23) = 0.49, *p* < .05] and were better than the clinical group at discriminating between safe and dangerous scripts [*t* (175) = -7.95, *p* < .001].
Berle & Moulds, (2013b)- Australia	Study 1: 70 (mean age = 19.09; SD = 2.14; 42 females, 60%) Case-Control study	Low dysphoric (*n* = 28) High dysphoric (*n* = 42)	ASI BDI-II DAS TAS	Study 1: - Task: Adaptation of Arntz et al., (1995). - Administration: Computer. - Number of scenarios: 7 (6 for dysphoria and 1 for panic attack). - Assessment: 100mm VAS (How unfortunate- negative- worthless- incompetent- hopeless- controllable is the situation?).	Study 1: - ER use was comparable in low and high dysphoric groups (all *p* > .05). - Ratings of how incompetent one was in each situation approached significance [*t* (1,68) = 2.27, *p* = .03]. - ER did not correlate with self-report measures of dysfunctional attitudes, anxiety sensitivity and alexithymic tendencies (all *p* > .05).
	Study 2: 118 (mean age = 19.79, SD = 4.94; 91 females, 77.1%) Case-Control study	Low dysphoric (*n* = 20) High dysphoric (*n* = 34) Low anxiety symptoms (*n* = 44) High anxiety symptoms (*n* = 34)	BAI BDI-II DAS WST	Study 2: - Same script as in Study 1. - Assessment: Modification of VAS to self-referent ratings (how pathetic and inadequate participants would consider themselves, and ratings of perceived worthlessness and incompetence), non-self-referent ratings (unfortunate and negative the situation was interpreted), and Anxiety rating (Dangerous).	Study 2: - Depression-related ER scores were comparable in high dysphoric and low dysphoric groups (all *p*> .05). - Three of the four self-referent ER ratings (incompetent [*r* = .23; *p* < .05], pathetic [*r* =.26; *p* < .05], and inadequate [*r* = .33; *p* < .05]) showed mild positive correlations with depressive symptoms. - No differences in the anxiety-related scenario (all *p* > .05) between the low and the high anxiety groups. - The low dysphoric group scored higher than the high dysphoric group in the dangerousness rating in all scenarios (all *p* < .05). - ER was stable (8-week interval; .53 ≤ r ≤ .74, *p* <.05), although not predictive of subsequent depressive symptoms.
Berle & Moulds, (2013a)- Australia	78. Age and sex not reported. Case-Control study	Currently depressed (*n* = 27) Currently non-depressed: Never depressed (*n* = 25) Previously but not currently depressed (*n* = 26)	SCID-I BAI BDI-II PHQ-9 SUIS WST (Modified version)	- Task: Adaptation of Arntz et al., (1995). - Administration: Computer. - Number of scenarios: 7 (6 for dysphoria and 1 for panic attack). - Assessment: Same as Berle & Moulds, (2013b).	- No differences in ER between depressed and non-depressed participants (all *p* > .05). - Compared with never- depressed participants, previously depressed engaged more frequently in two non-self-referent ER ratings: How unfortunate [*t* (75) = 2.20, *p* = .03] and how negative the situation was perceived to be [*t* (75) = 2.09, *p* = .04].
Berle et al., (2016)- Australia	36 (mean age = 32.6 Female = 26 -72.2%) Before-After (Pre-Post) Study with No Control Group	Co-occurring disorders: Major depressive episode (*n* = 18) Dysthymia (*n* = 7) Panic disorder with or without Agoraphobia (*n* = 7) Agoraphobia without Panic disorder (*n* = 4) Social anxiety disorder (*n* = 10) OCD (*n* = 4) PTSD (*n* = 5) GAD (*n* = 12) Specific phobia (*n* = 13) Hypochondriasis (*n* = 2) Alcohol abuse or dependence (*n* = 5)	MINI BAI BDI-II The Beliefs about Emotions Questionnaire CERQ SUIS Experimental ER Task	- Task: Adaptation of Arntz et al., (1995). - Administration: Paper and pencil. - Number of scenarios: 4 (2 for panic attacks and agoraphobia, 1 for GAD, and 1 for health anxiety). - Assessment: 100mm VAS including self-referent ratings (How incompetent does this situation suggest that you are), non-self-referent ratings (how likely is it that something bad will occur, how likely is it that you would be unable to cope, how bad is the worst possible outcome, how negative was the situation), and Anxiety rating (Dangerous).	- Pretreatment: When an anxious emotional response was presented, individuals seeking treatment for an anxiety disorder rated the situations as more dangerous [*t* (35) = 7.05, *p* < .001, *d* = 1.18] and negative [*t* (35) = 8.36, *p* < .0001, *d* = 1.39]. They were also more likely to experience that something bad would happen [*t* (35) = 6.86, *p* < .0001, *d* = 1.14], were more likely to feel unable to cope [*t* (35) = 7.64, *p* < .0001, *d* = 1.27], were more likely to feel incompetent in the situation [*t* (35) = 4.71, *p* < .0001, *d* = 0.79], and were more likely to estimate the worst possible result as bad [*t* (35) = 8.11, *p* < .0001, *d* = 1.35]. - Post-treatment: Anxiety (95% CI [4.60, 17.24]) and depression symptoms (95% CI [4.92, 13.64]) were reduced with the treatment. However, only one ER score (i.e., incompetence) significantly decreased after therapy (95% CI *t* [2.13, 13.24]).
Engelhard et al., (2001)- The Netherlands	30 (100% males). Age not reported. Case-Control study	PTSD (*n* = 15) No history of PTSD (*n* = 15)	Intrusion-based reasoning task (IR) (four PTSD-specific scenarios based on case reports of Vietnam War veterans) PSS-SR STAI-T ASI ISQ (modified) CES Shipley Institute of Living Scale	- Task: Adaptation of Arntz et al., (1995). - Administration: Telephone. - Number of scenarios: 3 (1 for panic disorder, social phobia, and spider phobia). - Randomly ordered. - Assessment: 100mm VAS (1 dependent variable: danger; and 4 additional outcomes: safety, uncontrollability, anxiety, and positive vs negative outcome.	- Veterans without PTSD only inferred the danger from scenarios with objective danger stimuli. Conversely, veterans with PTSD also inferred danger in the presence of non-objective scripts, including anxiety [*F*(1, 28) = 6.20, *p* = .019] and intrusions [*F*(1, 28) = 8.25, *p* = .008]. - ER and IR (defined as the difference in danger ratings between scenarios with and without information about anxiety and intrusions) were moderately, yet not significantly related [*r* (30) = 0.35, *p* = .055]. - ER was unrelated to negative appraisals of posttraumatic symptoms, anxiety sensitivity, trait anxiety, and intelligence (all *p* > .05).
Gangemi et al., (2007)- Italy	120 (mean age = 22.4). Sex not reported Case-Control study	Guilt group (*n* = 36) Anxiety group (*n* = 43) Neutral group (*n* = 41)	Trait and State Guilt Inventory State Guilt Inventory PANAS Affect induction	- Task: Adaptation of Arntz et al., (1995). - Administration: Paper and pencil. - Number of scenarios: 2. - Assessment: 100mm VAS (Likelihood and the severity of the negative event, and the dissatisfaction with the preventive action).	- Compared with low-trait guilt participants, people high in trait guilt had higher risk ratings after an induction of state guilt (likelihood: [*F* (2,114) = 3.2, *p*>.05, *d* = 0.45]; severity: [*F* (2, 114) = 4.8, *p* >.05, *d* = 0.66]). - Participant with high-trait guilt were more likely to respond intensely to state guilt [*F* (1, 114) = 24.6, *p* > .001, *d*= 1]. - The interaction between Affect Induction and Trait guilt did not reach statistical significance [*F*(2, 114) = 2.3, *p* = .1]. However, people with a general inclination to feel guilty were more likely to use temporary feelings of guilt as information about the threat content of a situation even if the source of state guilt was unrelated to the situation.
Lommen et al., (2013)- The Netherlands	58 (mean age = 22, SD = 2.56; 8 males) Follow-up: 31 Controlled Intervention Study	Fear of spiders. Control group (*n* = 29; 5 males) Experimental group (*n* = 29; 3 males) Follow-up: Control group (*n* = 17) Experimental group (*n* = 14)	FSQ Quiz including seven questions about spider-related facts STAI-1 ER Task (1-2) Computerized training STAI-2 BAT STAI-3 Follow-up assessment: BAT	- Administration of the training: Computer. - Number of scenarios: 60 (objective safe or danger information, with an anxious or non-anxious response, and an unfinished sentence to complete by participants). -Feedback differed between conditions. In the experimental condition, it depended on objective safe (positive outcome) or danger (negative outcome) information. In the control condition, feedback depended on non-anxious response (positive outcome), or anxious response (negative outcome) information.	- The manipulation significantly decreased ER in the experimental condition (M_diff_ = 12.65, *p* < .01, 95% CI [5.22, 20.08]), but not in the control condition (M_diff_ = 0.15, *p* = .97, 95% CI [–7.28, 7.58]). In the experimental condition, immediate reductions in the perceived danger of a spider also occurred [*F* (1, 53) = 6.79, *p* = .01, *d* = 0.70]. This effect was maintained the day after [*F*(1, 27) = 4.53, *p* = .04, *d* = 0.79]. - The approach behavior with a spider did not change immediately after the manipulation [*F*(1, 53) = 0.51, *p* = .48, *d* = 0.19] and one day later [*F*(1, 27) = 1.75, *p* = .20].
Verwoerd et al., (2013)- The Netherlands	164 (mean age = 22.62, SD = 7.12; female 70%) Case-Control study	Higher clinical range in contamination fear (*n* = 31) Low range in contamination fear (*n* = 27)	Padua Inventory	- Task: Adaptation of Arntz et al., (1995). - Administration: Online. - Number of scenarios: 8 (contamination and disgust response). - 32 scripts were distributed over 4 versions of the task. - Assessment: 100mm VAS (3 dependent variables: danger, risk of contamination, and risk of becoming ill; and 3 additional as filler)	- When a disgust response was presented, this led to a general overestimation of danger irrespective of the group [*F*(1, 56) = 5.80, *p* < .001, *d* = 0.66]. - Adding the disgust response information to low threat scenarios resulted in an overestimation of the risk of becoming ill [*t* (58) = 6.82, *p* < .001]. -The disgust-based emotional reasoning bias was especially evident in participants high in contamination fear [*F*(1, 56) = 4.05, *p* < .05, *d* = 0.548]. -A similar emotional reasoning bias was observed in high fearful participants when they had to infer danger or the risk of becoming contaminated.
Verwoerd et al., (2016)- The Netherlands	144 (mean age = 22.62, SD 1/4 7.12; female 77%) Case-Control study	High fear of vomiting (*n* = 35) Low fear of vomiting (*n* = 38)	EQ	- Task: Adaptation of Verwoerd et al., (2013). - Administration: Online. - Number of scenarios: 8 (fear of vomiting). - Assessment: Same as a Verwoerd et al., (2013).	- Participants generally used emotional response information (anxiety and disgust) to infer danger [*F*(1, 71) = 15.66, *p* < .001, *d* = 0.93], risk of contamination [*F*(1, 71) = 18.76, *p* < .001, *d* = 1.03], and risk of becoming ill [*F* (1, 71) = 33.19, *p* < .001, *d* = 1.37]. - The overestimation of contamination ratings based on disgust was stronger for disgust than for anxiety [*F*(1, 71) = 3.96, *p* = .05, *d* = 0.45]. - The impact of emotional response information on the subjective risk of becoming ill was especially evident in high vomit fearful participants [*F*(1, 71) = 5.87, *p* < .05, *d* = 0.58]. - Follow-up analyses taking into account shared variance between both disgust and anxiety revealed greater ER in the high vomit fearful group [χ^2^ (2) = 7.19, *p* = .03], which was mainly driven by the emotion of disgust (Wald = 4,47, *p* = .013).

Note: *ASI*, Anxiety Sensitivity Index; *BAI*, Beck Anxiety Inventory; *BA*T, Behavioural Approach Task; *BDI-II*, Beck Depression Inventory II; *CERQ*, Cognitive Emotion Regulation Questionnaire; *CES*, The Combat Exposure Scale; *DAS*, Dysfunctional Attitude Scale – Form A; *ER*, Emotional Reasoning; *EQ*, Emetophobia Questionnaire; *FSQ*, Fear of Spiders Questionnaire; *GAD*, Generalized anxiety disorder; *ISQ*, Interpretation of Symptoms- Questionnaire; *MINI*, Mini Neuropsychiatric Interview; *OCD*, Obsessive-compulsive disorder; *PANAS*, Positive and Negative Affect Scale; *PHQ-*9, Patient Health Questionnaire 9-item depression scale; *PSS-SR*, Posttraumatic Symptom Scale; *PTSD*, Posttraumatic stress disorder; *SCID-*I, Structured Clinical Interview for DSM-IV Axis I disorders non-patient edition; *SCL*-90, Symptom Checklist-90; *STAI-T*, State-Trait Anxiety Inventory; *SUIS*, Spontaneous Use of Imagery scale; *TAS*, The 20-item Toronto Alexithymia Scale; *VAS*, Visual Analogue Scale; *WST*, Wason Selection Task

### Risk of bias assessment

To assess study quality and risk of bias of the included articles we used the Study Quality Assessment Tools from the National Heart Lung and Blood Institute (National Heart Lung & Blood institute, 2020). Total quality scores could range between 0 and 13 points. Depending on the total score, this tool allows reviewers to rate studies as “good”, “fair”, or “poor”. The quality review of articles was carried out by two reviewers independently. When the reviewers disagreed, they met to discuss the case.

### Synthesis of results

The results extracted from the studies, which are presented in Table 1, were not analyzed statistically in a meta-analysis due to the heterogeneity of studies in terms of the diagnostics included, the designs implement, and measures administered, and analytical approaches used.

## Results

### Sample characteristics of the included studies

The characteristics of each of the 9 studies included in this systematic review are summarized in Table 1. Most of the investigations were conducted in The Netherlands (*n* = 5) (Arntz et al., 1995; Engelhard et al., 2001; Lommen et al., 2013; Verwoerd et al., 2013, 2016), followed by Australia (*n* = 3; Berle et al., 2016; Berle & Moulds, 2013a, b), and one study in Italy (Gangemi et al., 2007). Regarding the year of publication, the first article on ER was published in 1995 and the last one in 2016, so there have not been very recent advances on the topic. Most included participants have been young women, with an average age between 19 and 33 years.

Regarding the design of the investigations, the majority of articles (*n* = 7) were Case–Control studies (Arntz et al., 1995; Berle & Moulds, 2013a, b; Engelhard et al., 2001; Gangemi et al., 2007; Verwoerd et al., 2013, 2016). Overall, their main objective was to evaluate ER characteristics in participants with an emotional disorder without the administration of an intervention. One additional study was a Controlled Intervention which aimed to evaluate the efficacy of a computerised experimental training against a control training to decrease ER (Lommen et al., 2013). The remaining investigation was a Before-After (Pre-Post) study with No Control Group. Its goal was to determine whether ER tendencies changed during a course of a cognitive-behavioral psychotherapy intervention (Berle et al., 2016).

The majority of investigations focused on anxiety disorders only (*n* = 6; Arntz et al., 1995; Engelhard et al., 2001; Gangemi et al., 2007; Lommen et al., 2013; Verwoerd et al., 2013, 2016) and included patients with a wide range of disorders, such as Spider Phobia, Panic disorder, Social Anxiety Disorder, Obsessive–Compulsive Disorder, Generalized Anxiety Disorder, and Posttraumatic Stress Disorder. These studies evaluated a total of 698 participants with anxiety disorders. Two studies (*n* = 2) evaluated patients with depression or dysthymia (Berle & Moulds, 2013a, b). These included a total of 202 participants. The remaining investigation included individuals with comorbid diagnoses (Berle et al., 2016), both in the spectrum of anxiety and depression disorders (e.g., Depression, Dysthymia, Panic, Social anxiety, Specific phobia, etc.).

### Data analysis

For the case–control studies (Arntz et al., 1995; Berle & Moulds, 2013a, b; Engelhard et al., 2001; Gangemi et al., 2007; Verwoerd et al., 2013, 2016), *the authors reported a* comparison of means according to *the* assessment scale, as well as a correlation between the ER measure and measures of psychopathology. In the Controlled Intervention (Lommen et al., 2013) and the before-after (pre-post) study without control group (Berle et al., 2016), the *authors presented a cross-group* comparison of ER means *across different time periods*.

### Measures used

#### Measurement of clinical characteristics

The included studies used a wide range of measures depending on their goals. For the evaluation of clinical symptoms, the authors used the Structured Clinical Interview for the DSM-III-R (Arntz et al., 1995; Berle & Moulds, 2013b), the SCL-90 (Arntz et al., 1995) or the Mini Neuropsychiatric Interview (Berle et al., 2016). For the assessment of anxiety, the most frequent tools were the Anxiety Sensitivity Index (Berle & Moulds, 2013a; Engelhard et al., 2001), the Beck Anxiety Inventory (Berle & Moulds, 2013a, b; Berle et al., 2016), and the Trait scale of the State-Trait Anxiety Inventory (Engelhard et al., 2001; Lommen et al., 2013). To evaluate depressive symptoms, studies either administered the Beck Depression Inventory II (Berle & Moulds, 2013a, b; Berle et al., 2016) or the Patient Health Questionnaire 9 (Berle & Moulds, 2013b). In addition to these, some investigations evaluated specific symptomatology, such as fear of contamination (Padua Inventory; Verwoerd et al., 2013), guilt (Trait and State Guilt Inventory; Gangemi et al., 2007), or fear of vomiting (Emetophobia Questionnaire; Verwoerd et al., 2016)). When posttraumatic stress was the focus, Engelhard et al. (2001) also administered the Posttraumatic Symptom Scale, the Interpretation of Symptoms-Questionnaire, the Combat Exposure Scale, and the Shipley Institute of Living and Scale.

#### Measurement of emotional reasoning

The measure developed by Arntz et al. (1995) has been taken as the reference tool to evaluate the presence of ER in patients with emotional disorders. It consists of 16 scripts, each followed by 100 mm visual analogue scales (VAS). In the original version, the authors evaluated 4 areas, spider phobia, panic disorder, social phobia, and a control situation. The main characteristic of the scripts is that they all start identically (e.g., "You are in the elevator in the largest department store in Maastricht, intending to take it from the fifth to the first floor. Breathing is getting more difficult. The elevator is packed with the maximum number of people allowed."). The interesting point is that, for each area, there are 4 versions of how the basic script ends: 1. Version one has objective safety information and a non-anxious response (e.g., "One of the passengers accidently falls into your arms. You smile. You have been interested in this person for quite some time and this seems to be a good opportunity.”); 2. Version two includes objective safety information and an anxious response (e.g., "Suddenly you become very anxious."); 3. The third ending incorporates objective danger information and a non-anxious response (e.g., "All of the sudden the elevator gets stuck between floors. The ventilator stops and the elevator will not budge. You see two people faints: one falls into your arms. You smile. You've been interested in this person for quite some time, and this seems to be a good opportunity."); 4. The fourth and final version presents objective danger information and an anxious response (e.g., "All of the sudden the elevator gets stuck between floors. You have seen two people faints. Suddenly you become very anxious.").

Since the creation of the scenarios by Arntz et al. (1995), consecutive authors have made modifications to the scenarios to adapt them to specific disorders, such as different themes, the number of scenarios or different administration methodologies (e.g., onsite by pencil and paper, by telephone, or online).

One of the Case–Control studies implemented the original scenarios by Arntz et al. (1995) with no further modifications (Engelhard et al., 2001). However, most investigations adapted the scenarios to be used in specific populations. For example, Gangemi et al. (2007) adapted the scenarios for their use in Obsessive Compulsive Disorder, while Verwoerd et al. (2013, 2016) adapted them to evaluate emetophobia and fear of contamination. None of the authors, however, made significant modifications to the VAS scales and maintained the measurement of “dangerousness” in the response options.

The largest changes occurred in the studies where depression and dysthymia were the focus (Berle & Moulds, 2013a, b). Here, not only the scenarios were modified to be adapted to the symptomatology, but also the VAS scales were changed to evaluate self-reference (e.g., how pathetic and inadequate participants would consider themselves) and non-self-reference ratings (e.g., how unfortunate and negative the situation was interpreted to be). This was made because, in depression and related disorders, ER is thought to impact the person's assessment of their own performance to a greater extent than the assessment made by external individuals (Berle & Moulds, 2013a, b).

### Main findings of the included studies

As summarized in Table 1, studies that evaluated persons with anxiety problems (Arntz et al., 1995; Engelhard et al., 2001; Gangemi et al., 2007; Verwoerd et al., 2013, 2016) supported the importance of ER in this sample. Particularly, when comparing persons with significant clinical symptoms and controls (persons without symptoms or with less severe symptomatology), group differences emerged particularly when no objective danger information was present. Specifically, the persons with anxiety problems perceived scenarios as more dangerous than persons without the problem when no objective danger information was present, which is reflects more ER in the former. Thus, the anxious group had more difficulties when discriminating between safe and dangerous situations (Arntz et al., 1995). The studies also revealed that the severity of the anxiety symptomatology was associated with the severity of ER. A study also showed that these findings were robust in time (Verwoerd et al., 2016).

Different to studies including persons with anxiety disorders, the two studies exploring the role of ER in persons with depression and dysthymia (Berle & Moulds, 2013a, b) failed to reveal significant differences in ER scores between the comparison groups. There was only one exception to this: participants with depression engaged in more non-self-referent ER than never-depressed participants (Berle & Moulds, 2013b). Note, however, that self-referential ER measurements were only weakly correlated with the severity of depressive symptoms and uncorrelated with the remaining self-report measures, that is, dysfunctional activities, anxiety, and alexithymia.

In the study by Berle et al. (2016), patients who sought treatment and began cognitive behavioral psychotherapy had high ER bias scores. This led them to score the situations that presented an anxious response as more dangerous. After the treatment, only a reduction in one ER rating, namely “Incompetence” was observed. The remaining ratings (“Dangerousness”, “negativity”, “likelihood of something bad happening”, “likelihood of not being able to cope”, and “worst outcome”) showed no significant difference in the pre-to-post treatment comparison (Berle et al., 2016).

A final study conducted by Lommen et al. (2013) intended to modify ER bias using a computerised training. The authors demonstrated associating positive feedback to scenarios that included objective safety information had a positive influence on ER, even when compared to the control condition. These changes had, in turn, a positive impact on the reduction of danger to spiders and the benefits were maintained at follow-up (i.e., one day later). In this study, the manipulation consisted of a computer-based training with 60 scenarios in which each participant had to imagine a situation as if it had happened to them. In the task, they had to complete each sentence with either a positive or a negative outcome. Next, to reduce ER in the experimental condition, scenarios with objectively safe information had a positive outcome, while a scenario with objectively dangerous information had a negative outcome. In this way, participants could learn that the objective information was the only relevant information that predicted the outcome, regardless of the emotional response. For this, feedback was given based on their choice (i.e., "correct" or "incorrect, try again").

### Limitations in the included studies

The included studies generally share several limitations. On the one hand, they all tend to include subclinical samples, frequently students (*n* = 5;Berle & Moulds, 2013a, b; Gangemi et al., 2007; Verwoerd et al., 2013, 2016). They also tend to include small sample sizes (Berle et al., 2016; Lommen et al., 2013), which puts into question the generalizability and robustness of the findings. Another shortcoming lies in the heterogeneity of diagnosis, which was argued to negatively impact the generalizability of findings in the Before-After study by Berle et al. (2016). Additionally, Lommen et al. (2013) referred to the brevity of the training conducted and the follow-up period (i.e., only), as well as to the multiplicity of the thematic ER scenarios included as limitations in their study.

On the other hand, the authors in the presented studies generally note that the scenarios developed have not been systematically validated and may lack ecological validity. That is, the effects of ER in real life may not correspond to those of imaginary situations based on a scenario. The types of assessments used also make it impossible to generate causal inferences.

### Risk of bias assessment

The evaluation of the risk of bias in the included studies was carried out following the Study Quality Assessment Tools from the National Heart Lung and Blood Institute (National Heart Lung & Blood institute, 2020). According to these guidelines, we classified the studies into 3 categories (see Tables 2, 3 and 4): Case–Control Studies (*n* = 7), Before-After (Pre-Post) Studies with No Control Group (*n* = 1), and Controlled Intervention Studies (*n* = 1).

**Table 2:** Quality assessment of case–control studies

Reference	1. Research question	2. Study population	3. Sample size justification	4. Groups recruited from the same population	5. Inclusion and exclusion criteria prespecified and applied uniformly	6. Case and control definitions	7. Random selection of study participants	8. Concurrent controls	9. Exposure assessed prior to outcome measurement	10. Exposure measures and assessment	11. Blinding of exposure assessors	12. Statistical analysis	Total score (maximum 12 points)
Arntz et al. (1995)	Yes	Yes	No	No	NR	Yes	Yes	NR	Yes	Yes	NR	Yes	7
Berle et al. (2013a)	Yes	Yes	No	Yes	Yes	Yes	NA	NR	Yes	Yes	NR	Yes	8
Berle et al. (2013b)	Yes	No	Yes	Yes	Yes	Yes	NA	NR	Yes	Yes	NR	Yes	8
Gangemi et al. (2007)	Yes	Yes	No	Yes	Yes	Yes	Yes	NR	Yes	Yes	NR	Yes	9
Engelhard et al. (2001)	Yes	No	No	NR	Yes	Yes	NA	NR	Yes	Yes	Yes	Yes	7
Verwoerd et al. (2013)	Yes	Yes	No	Yes	Yes	Yes	NA	NR	Yes	Yes	NR	Yes	8
Verwoerd et al. (2016)	Yes	Yes	No	Yes	Yes	Yes	NA	NR	Yes	Yes	NR	Yes	8

*NR* Not Reported

**Table 3:** Quality assessment tool for before-after (Pre-Post) studies with no control group

Reference	1. Study question	2. Eligibility criteria and study population	3. Study participants representative of clinical populations of interest	4. All eligible participants enrolled	5. Sample size	6. Intervention clearly described	7. Outcome measures clearly described, valid, and reliable	8. Blinding of outcome assessors	9. Followup rate	10. Statistical analysis	11. Multiple outcome measures	12. Group-level interventions and individual-level outcome efforts	Total score (maximum 12 points)
Berle et al. (2016)	Yes	Yes	No	Yes	No	No	Yes	NR	No	Yes	No	No	6

*NR* Not Reported

**Table 4:** Quality assessment of controlled intervention studies

Reference	1. Described as randomized	2. Method of randomization	3. Treatment allocation concealed	4. Blinding- Study participants and providers	5. Blinding- People assessing	6. Similarity of groups at baseline	7. Overall Dropout 20% or lower	8. Differential Dropout 15% or lower	9. Adherence	10. Avoid other interventions	11. Outcome measures assessment	12. Power calculation	13. Prespecified outcomes	14. Intention-to-treat analysis	Total score (maximum 14 points)
Lommen et al. (2013)	No	No	Yes	NR	No	Yes	No	No	Yes	Yes	Yes	Yes	No	No	6

*NR* Not Reported

Case–Control Studies (Arntz et al., 1995; Berle & Moulds, 2013a, b; Engelhard et al., 2001; Gangemi et al., 2007; Verwoerd et al., 2013, 2016) had a score between 7 and 9 out of a total of 12 points. Regarding sample size justification, only one study justified that their sample size was considered sufficient (Berle & Moulds, 2013b). In addition, five of the studies (Berle & Moulds, 2013a, b; Engelhard et al., 2001; Verwoerd et al., 2013, 2016) did not apply a random selection to the participants, as the study was conducted with the entire sample collected. Two of the risk of bias assessment points, namely “Concurrent controls” and “Blinding of exposure assessors” were not detailed in any of the included studies.

The Before-After (Pre-Post) Study with No Control Group (Berle et al., 2016) obtained a total of 6 points out of 12. It had a small sample that was not representative of the population, they did not collect 80% of the participants for the follow-up, and the intervention was not clearly explained.

Finally, the Controlled Intervention Study (Lommen et al., 2013) only reached a score of 6 out of 14 points. It was not described as randomized, so it did not use methods such as sample randomization or blinding participants and providers to the assigned treatment group. In addition, dropout was higher than 20% and outcomes were not prespecified.

## Discussion

This study aimed to systematically review the literature into the role of ER in anxiety and depression disorders, particularly the extent to which ER might be related to the occurrence and severity of emotional disorders. A thorough search resulted in 9 original studies included in the synthesis. In general, most of them have been case–control studies (*n* = 7), have included a variety of emotional disorders, mostly anxiety-related disorders, and have obtained moderate quality evaluations. Overall, the findings support the utility of ER for the discrimination of persons with and without anxiety disorder and to quantify the severity of such disorder, but findings are less promising in the case of depression disorders. As we will detail in the next lines, the present systematic review evidences the need for higher quality research using larger samples, conducting cross-cultural comparisons, developing intervention studies in a wide range of emotional disorders and using methodologically-sound measures.

First, interest in the field of ER in emotional disorders has fluctuated very significantly during the last decades. Since the first publication in 1995, the following two studies did not occur until 6 and 12 years later, respectively. It was not until 2013 when the interest in this topic reached a stable peak and six studies were published from 2013 to 2016. Surprisingly, though, no investigation on the topic has appeared in the past 5 years. Also importantly, studies have been generally conducted in a few countries (The Netherlands and Australia) by the same groups of researchers. Overall, this supports the need for further research, with special focus on cross-cultural publications. The effect of culture on mental health has been demonstrated. For example, emotional suppression, which is a risk factor for mental and physiological illnesses and poor social and psychological adjustment, is favored in collectivist cultures (Ford & Mauss, 2015; Liddell & Williams, 2019; Ramzan & Amjad, 2017). The role of ER as a regulatory strategy in different cultures, however, remains unexplored. Future efforts should be conducted to explore whether ER is more prevalent in certain cultures (e.g., collectivist vs individualist) and to what extent the influence of ER on psychological wellbeing depends on cultural differences.

Another interesting finding was that most research has been conducted with young females and persons with anxiety disorders. Emotional disorders are indeed more frequent in females and young adults (Kessler et al., 2005; Li & Graham, 2017; Rubinow & Schmidt, 2019). However, the excessive focus on young women and the small sample sizes in the majority of studies has made an analysis of sex/gender and age differences in the role of ER impossible. These analyses are frequent in similar fields, such as emotion regulation, and have generally shown both sex/gender and age differences. For example, women seem to report a more frequent use of both maladaptive and adaptive strategies compared to men, and the use of these strategies decreases with age (Nolen-Hoeksema & Aldao, 2011; Zimmermann & Iwanski, 2014). The extent to which this is also true for ER remains unclear, so future studies should prioritize this type of analyses to provide evidence that guides interventions in a more efficient manner (i.e., personalized treatments).

Of the 9 articles included in the present study, 7 focused on the assessment of ER. Only two studies implemented an intervention to investigate whether changes in ER could be achieved with psychotherapy. Regarding these intervention studies, a computerised ER training resulted in significant reductions in ER (Lommen et al., 2013). Another study, however, indicated much more modest findings, as evidenced by a slight reduction in only one ER self-referent rating (e.g., How incompetent does this situation suggest that you are?) out of 6 outcomes after a CBT-based treatment (Berle et al., 2016). This reduced number of studies make it difficult to establish the efficacy of psychological treatments for ER reductions. Also importantly, the extent to which changes in ER lead to improved outcomes in persons with emotional disorders, such as reduced depression or anxiety (i.e., mediation), remains unexplored. Therefore, the literature needs more quality clinical trials that explore whether a) ER can be effectively changed with psychotherapy and b) the extent to which such changes in ER are responsible for the improvements in outcomes in persons with emotional disorders (i.e., mediation research). Only after robust evidence in this line is obtained, ER bias modification will be an appealing training component to be included in multicomponent psychological interventions for emotional disorders.

Another finding in the present review was that most studies into ER have focused on anxious symptomatology as opposed to depressive symptoms. These studies have generally supported that ER might be underlying processes of anxiety disorders. In particular, it seems that people with anxiety-related symptoms tend to infer danger not only on the basis of the presence of objective danger, but also on the basis of a subjective anxiety reaction (i.eArntz et al., 1995; Berle & Moulds, 2013b). Ultimately, this bias may play an important role in people's predisposition to develop and maintain anxiety-related disorders. If systematically replicated cross-culturally and across age and sex/gender groups, this could be especially relevant for preventive and treatment programs for anxiety-related disorders.

In contrast to anxiety research, the study of ER in depression has been rare and has failed to support to the role of ER in this type of symptomatology. For example, Berle and Moulds (2013b) demonstrated that participants with depression scored only mildly and non-significantly higher in ER when compared with participants without depression. Problems with the assessment instruments (e.g., lack of construct validity in patients with depression and no ecological validity) or the limited number of investigations (*n* = 2) might partly explain the discrepancy in the role of ER when comparing anxiety and depression problems. However, it is also possible that differences in the processes underlying depression and anxiety problems exist. In this sense, Berle and Moulds (2013b) suggest that anxious individuals may remain vigilant about both their external and internal signals (including their emotional states) for signs of imminent threat. This was confirmed, for example, in the case of persons with posttraumatic stress disorder (Engelhard et al., 2001), who tend to develop anxious apprehension about anxiety and associated traumatic memories. A similar finding has been reported in persons with high trait guilt. These individuals tend to use feelings of guilt as sources of information when assessing threat, which helps explain the development, maintenance, and aggravation of obsessive–compulsive disorders (Gangemi et al., 2007). Different to that, it is possible that participants with depression tend to be detached from present state stimuli as their attention and thinking remain focused on past losses, failures, and enduring inadequacies (Berle & Moulds, 2013a, b). If anxious individuals have a greater awareness of emotional states than participants with depression, then they are more likely to allow emotions to influence their interpretations of situations and therefore behave according to a bias in ER (Berle & Moulds, 2013b). These findings would call into question the idea of ER as a transdiagnostic process (Harvey et al., 2004, 2011), which argued that elevated levels of ER characterized both anxiety and depressive disorders. While acknowledging this, further investigation with robust and well-validated ER measures is still needed to determine whether ER should be considered or ignored in psychological treatments for depression.

An interesting finding in the present review was that all the included ER studies have followed the same model for the assessment of ER biases. Specifically, they have described scenarios with 4 different endings (objective safety information and non-anxious response, objective safety information and anxious response, objective danger information and non-anxious response, and objective danger information and an anxious response). This combination of alternative endings appears to be key to obtain a reliable and valid test of ER that allows to evaluate an individual’s appraisal in different situaions. Importantly, all the studies conducted in persons with anxiety-related disorders utilized the same 100 mm VAS scale to evaluate each scenario (i.e., dangerous, safety, control, unpleasantness, and good vs bad expected outcome), which makes cross-diagnostic comparisons easier. The differences across measures occurred in the descriptions of the scenarios, which were adapted to the target population (e.g., panic disorder, obsessive–compulsive disorder, or posttraumatic stress disorder). Different to this, the studies that included participants with depression or dysthymia not only modified the description of the scenarios, but also changed the VAS scale for each of them. This allowed both the scenarios and the emotions assessed to be consistent with the population of interest and the main problems face by them. For example, in the case of anxiety problems, the VAS scales evaluated typical problems of persons with anxiety, such as danger, safety, control, unpleasantness, and outcome expectancy. On the contrary, when including persons with depression, the VAS scales evaluated self-referent ratings (e.g., feelings of inadequacy or incompetence in that scenario) and non-self-referent aspects (how unfortunate and negative the situation was), which are more characteristic of this population.

Another aspect that might have influenced the differences found between anxiety and depression studies into ER lies in the combination between the type of assessment and the differences in cognitive functions between both populations. Depression is known to have important consequences on cognitive functions including attention, visual learning, memory, and executive functioning (Lee et al., 2011), which might affect task performance. The ER tasks developed to date rely on the individuals’ cognitive abilities (e.g., attention, imagination, visualization, and decision-making). Therefore, it is possible that including images, videos, or virtual reality instead of written descriptions of scenarios only might facilitate the task of measuring ER in this population. Another important aspect that has not been analyzed so far is the importance of the ecological momentary assessment of ER, as this has been evaluated as a static trait only. There is now evidence that emotional regulation changes within and across days and that an ecological momentary system has the potential to capture this dynamic during the flow of daily experiences in real-life settings (Colombo et al., 2020). Therefore, future investigations should also consider evaluating ER in real-life contexts in a momentary and repeated manner.

## Limitations

The main shortcoming of the study lies in the limited number of studies found on ER and the heterogeneity of such studies in terms of sample size (from 30 to 164 participants), the type of study conducted (control-cases and one before-after investigation and a controlled intervention study), methodology used to assess ER (different VAS scales, different settings and different applications), and the instruments used to measure specific ER symptoms. These implications made a meta-analysis impossible. While acknowledging the valuable information that case–control and before-after studies provide, there is a need to conduct more methodically robust and ambitious studies that favor more convincing findings. This is also justified by the results obtained by our assessment of risk of bias. Finally, it is important to note that the results of this review may be biased due to the selection of databases and keywords and the interpretations made by the authors. The current guidelines on systematic review were followed to minimize such risk of bias (PRISMA; Page et al., 2021).

## Conclusions

Our results support the idea that ER is a transdiagnostic construct involved in the processes related to the development of anxious-related disorders, such as Spider Phobia, Panic disorder, Generalized Anxiety Disorder, Obsessive–Compulsive Disorder, and Posttraumatic Stress Disorder. More research is needed to confirm or disconfirm the idea that ER also underlies the onset and maintenance of depressive disorders. Recommendations on future research include: higher quality research using larger samples, cross-cultural comparisons, intervention studies in a wide range of emotional disorders, use of methodologically-sound measures, implementation of ecological momentary assessment, use of images, videos, or virtual reality for the assessment of the ER, and a combination of case–control and especially treatment trials that evaluate the mediating role of ER after psychological treatment.

## Data Availability

All data generated or analysed during this study are included in this published article.

## Appendix 1 Search term list

To conduct the search, the following terms were used together with Boolean operators such as OR or AND, giving as a result: ((“Emotional Reasoning”) OR (“ex-consequentia reasoning”) OR (“Affect-as-information”)) AND ((“emotional disorders”) OR (“anxiety”) OR (“depression”) OR (“depressive”)).

Search according to the database:

PUBMED:

("Emotional Reasoning"[All Fields] OR "ex-consequentia reasoning"[All Fields] OR "Affect-as-information"[All Fields]) AND ("emotional disorders"[All Fields] OR "anxiety"[All Fields] OR "depression"[All Fields] OR "depressive"[All Fields] OR "insomnia disorder"[All Fields] OR "borderline personality disorder"[All Fields] OR "BPD"[All Fields] OR "persistent complex bereavement disorder"[All Fields] OR "obsessive compulsive disorder"[All Fields] OR "OCD"[All Fields] OR "posttraumatic stress disorder"[All Fields] OR "PTSD"[All Fields] OR "dissociative disorders"[All Fields] OR "eating disorders"[All Fields] OR "selective mutism"[All Fields] OR "specific phobia"[All Fields] OR "social anxiety"[All Fields] OR "panic disorder"[All Fields] OR "agoraphobia"[All Fields] OR "generalized anxiety"[All Fields] OR "psychological disorder"[All Fields] OR "psychiatric disorder"[All Fields]).

SCOPUS:

TITLE-ABS-KEY ((("Emotional Reasoning") OR ("ex-consequentia reasoning") OR ("Affect-as-information")) AND (("emotional disorders") OR ("anxiety") OR ("depression") OR ("depressive") OR ("insomnia disorder") OR ("borderline personality disorder") OR ("BPD") OR ("persistent complex bereavement disorder") OR ("obsessive compulsive disorder") OR ("OCD") OR ("posttraumatic stress disorder") OR ("PTSD") OR ("dissociative disorders") OR ("eating disorders") OR ("selective mutism") OR ("specific phobia") OR ("social anxiety") OR ("panic disorder") OR ("agoraphobia") OR ("generalized anxiety") OR ("psychological disorder") OR ("psychiatric disorder"))).

THE COCHRANE LIBRARY

((("Emotional Reasoning") OR ("ex-consequentia reasoning") OR ("Affect-as-information")) AND (("emotional disorders") OR ("anxiety") OR ("depression") OR ("depressive") OR ("insomnia disorder") OR ("borderline personality disorder") OR ("BPD") OR ("persistent complex bereavement disorder") OR ("obsessive compulsive disorder") OR ("OCD") OR ("posttraumatic stress disorder") OR ("PTSD") OR ("dissociative disorders") OR ("eating disorders") OR ("selective mutism") OR ("specific phobia") OR ("social anxiety") OR ("panic disorder") OR ("agoraphobia") OR ("generalized anxiety") OR ("psychological disorder") OR ("psychiatric disorder"))) in Title Abstract Keyword—(Word variations have been searched).

PSYC INFO

(("Emotional Reasoning") OR ("ex-consequentia reasoning") OR ("Affect-as-information")) AND (("emotional disorders") OR ("anxiety") OR ("depression") OR ("depressive") OR ("insomnia disorder”) OR (“borderline personality disorder”) OR (“BPD”) OR (“persistent complex bereavement disorder”) OR (“obsessive compulsive disorder”) OR (“OCD") OR (“posttraumatic stress disorder”) OR (“PTSD”) OR ("dissociative disorders") OR ("eating disorders") OR (“selective mutism”) OR (“specific phobia”) OR (“social anxiety”) OR (“panic disorder”) OR (“agoraphobia”) OR (“generalized anxiety”) OR ("psychological disorder") OR (“psychiatric disorder”))—in All Fields
